# Truck model recognition for an automatic overload detection system based on the improved MMAL-Net

**DOI:** 10.3389/fnins.2023.1243847

**Published:** 2023-08-10

**Authors:** Jiachen Sun, Jin Su, Zhenhao Yan, Zenggui Gao, Yanning Sun, Lilan Liu

**Affiliations:** ^1^Shanghai Key Laboratory of Intelligent Manufacturing and Robotics, School of Mechatronic Engineering and Automation, Shanghai University, Shanghai, China; ^2^College of Information Engineering, Lanzhou University of Finance and Economics, Lanzhou, China

**Keywords:** overload detection, truck model recognition, automatic weighing station, fine-grained visual categorization, MMAL-Net

## Abstract

Efficient and reliable transportation of goods through trucks is crucial for road logistics. However, the overloading of trucks poses serious challenges to road infrastructure and traffic safety. Detecting and preventing truck overloading is of utmost importance for maintaining road conditions and ensuring the safety of both road users and goods transported. This paper introduces a novel method for detecting truck overloading. The method utilizes the improved MMAL-Net for truck model recognition. Vehicle identification involves using frontal and side truck images, while APPM is applied for local segmentation of the side image to recognize individual parts. The proposed method analyzes the captured images to precisely identify the models of trucks passing through automatic weighing stations on the highway. The improved MMAL-Net achieved an accuracy of 95.03% on the competitive benchmark dataset, Stanford Cars, demonstrating its superiority over other established methods. Furthermore, our method also demonstrated outstanding performance on a small-scale dataset. In our experimental evaluation, our method achieved a recognition accuracy of 85% when the training set consisted of 20 sets of photos, and it reached 100% as the training set gradually increased to 50 sets of samples. Through the integration of this recognition system with weight data obtained from weighing stations and license plates information, the method enables real-time assessment of truck overloading. The implementation of the proposed method is of vital importance for multiple aspects related to road traffic safety.

## 1. Introduction

With the rapid development of the global economy and the acceleration of urbanization processes, highways play a crucial role in connecting different regions and cities. In the realm of road transportation, trucks serve as vital transportation tools, undertaking the task of transporting a substantial amount of goods. However, the issue of truck overloading has become one of the primary challenges in road traffic safety and road damage. Overloaded trucks exert significant pressure on road infrastructure, increasing the risk of traffic accidents and potentially leading to severe road collapse incidents. Therefore, the development of an accurate and efficient truckload monitoring method holds significant practical significance.

Traditional methods for truckload monitoring mainly rely on static weight measurement equipment such as weighbridges (Zhou et al., 2005) and fixed scales. However, these devices have several limitations, including the need for trucks to stop for measurement and high time and labor costs. Additionally, static measurement methods cannot provide real-time monitoring and detection capabilities for violations, limiting their effectiveness in practical applications.

To address these challenges, a promising solution has emerged: utilizing camera images from highway weigh stations for truck model recognition and combining them with weighing information obtained from a dynamic weighing system. By leveraging truck photos captured near these stations and employing advanced image processing and pattern recognition techniques, truck models can be identified accurately. Regrettably, the current network architectures utilized for recognition in this context often exhibit a simplistic nature, leading to suboptimal accuracy in the identification process. As a consequence, determining whether a truck is overloaded becomes inaccurate. Moreover, the ability to perform real-time recognition using captured photographs poses an unresolved challenge that demands urgent attention.

This paper aims to propose a truck model recognition method based on highway automatic weighing station camera images, with the objective of accurately identifying truck models. Consequently, the maximum load capacity of the trucks is determined. Through the integration of license plates and weighing information, the system can accurately determine if a truck is carrying excessive load. By doing so, it can prevent the occurrence of misjudgments caused by incidents of license plates damage, which are likely to happen in schemes that rely solely on license plates recognition to obtain vehicle models. Through this method, precise truck information can be provided for freight management, transportation safety, and highway planning, promoting the development of the logistics industry and enhancing traffic safety.

## 2. Literature review

### 2.1. FGVC

Fine-Grained Visual Categorization (FGVC) refers to the task of classifying objects into different subcategories or fine-grained classes within a broader category. In FGVC, the goal is to achieve detailed discrimination and classification among visually similar objects, such as different species of birds, breeds of dogs, or models of cars. This field of research focuses on developing computer vision algorithms and techniques to accurately recognize and classify objects at a fine-grained level, where subtle differences between subclasses need to be distinguished.

Fine-grained image classification, in contrast to conventional image classification tasks, encompasses a low signal-to-noise ratio, restricting the presence of highly discriminating information to minuscule local regions. Thus, the crux of achieving success in fine-grained image classification algorithms lies in the identification and efficient utilization of these valuable local region insights. Presently, most classification algorithms adhere to a common workflow: initial localization of the foreground object and its distinct local regions, followed by individual feature extraction from these regions. The processed features are subsequently utilized for classifier training and prediction purposes. To attain satisfactory classification results, numerous existing algorithms heavily depend on manual annotation information (Wei et al., 2018), such as bounding boxes and part locations. The annotation frame aids in foreground object detection, effectively mitigating background noise interference. Local region positions serve to identify valuable regions or align perspectives, facilitating the extraction of local features. Nevertheless, the costly acquisition of manual annotation information severely limits the practicality of these classification algorithms. In recent years, an increasing number of studies have opted to exclude such labeling information, relying solely on labels to accomplish image classification tasks (Lin et al., 2005; Zhang et al., 2016), resulting in commendable outcomes.

In the research and development of FGVC, traditional classification algorithms based on handcrafted features were initially employed. These algorithms typically begin by extracting local features, such as Histogram of Oriented Gradients (HOG) (Dalal and Triggs, 2005), from the images. Subsequently, an encoding model like Vector of Locally Aggregated Descriptors (VLAD) (Jégou et al., 2010) is employed for feature encoding, resulting in the desired feature representation. However, the limited descriptive power of handcrafted features often leads to suboptimal classification performance. In the early stages of fine-grained visual categorization research, the representation capacity of features became a primary bottleneck hindering performance improvement.

In recent years, Convolutional Neural Network (CNN)-based methods for fine-grained image recognition (Wang et al., 2017; Xie et al., 2017) have significantly matured. Donahue et al. (2014) conducted an analysis of a CNN model trained on the ImageNet dataset, revealing that the features extracted from the CNN possess more robust semantic characteristics and exhibit superior differentiation compared to artificial features. Building upon these findings, the researchers applied the convolution features to various domain-specific tasks, including fine-grained classification, resulting in improved classification performance. Nevertheless, the crucial components of the task tend to be subtle and were not adequately captured by conventional CNN approaches. Consequently, researchers have directed their attention toward internal enhancements within the framework. Zhang et al. (2014) introduced the Part R-CNN algorithm, which leverages R-CNN (Girshick et al., 2014) for image detection. This methodology aims to achieve precise localization of crucial components and enhance feature representation. Branson et al. (2014) proposed the Pose Normalized Convolutional Neural Network (Pose Normalized CNN) algorithm. Their approach comprises several steps: localization detection is performed on local regions for each input image, followed by cropping the image based on the detected annotation boxes, extracting hierarchical local information, and conducting pose alignment. Subsequently, distinct layers of convolutional features are extracted for different body parts. Finally, these convolutional features are concatenated into a feature vector and utilized for SVM model training. These approaches have demonstrated robust feature representation capabilities and yielded promising results in fine-grained image recognition tasks.

Compared to regular classification tasks, acquiring fine-grained image databases poses greater challenges and requires stronger domain expertise for data collection and annotation. However, in recent years, there has been a significant increase in the availability of fine-grained image databases, which reflects the flourishing development trend and strong real-world demand in this field. Currently, commonly used fine-grained image databases include (1) CUB200-2011: It comprises a total of 11,788 bird images belonging to 200 different categories. This database provides rich manual annotations, including 15 local part locations, 312 binary attributes, 1 bounding box, and semantic segmentation images, (2) Stanford Dogs: This database offers a collection of 20,580 images featuring 120 different breeds of dogs. It provides only bounding box annotations, (3) Oxford Flowers: This database is divided into two scales, containing 17 and 102 categories of flowers, respectively. The 102-category database is more commonly used, with each category containing 40 to 258 images. In total, there are 8,189 images in this database, which provides only semantic segmentation images without any additional annotations, (4) Cars: This database provides a collection of 16,185 vehicle images belonging to 196 different categories, encompassing various brands, years, and models. Only bounding box annotations are provided, and (5) FGVC-Aircraft: This database consists of 10,200 images of 102 different aircraft categories, with each category containing 100 distinct photos. Only bounding box annotations are provided. In recent years, extensive research has been conducted on fine-grained image databases. DCL (Chen et al., 2019) employed a deconstruction and reconstruction approach to learn semantic correlations among local regions in input images. API-Net (Zhuang et al., 2020) progressively recognized pairs of fine-grained images through iterative interaction. GCP (Song et al., 2022) introduced a dedicated network branch to magnify the importance of small eigenvalues. MSHQP (Tan et al., 2022) effectively modeled intra and inter-layer feature interactions, integrating multi-layer features to enhance part responses. These methods primarily focus on locating and utilizing key regions for final recognition, yielding promising performance. However, they tend to overlook the potential contribution of complementary regions that can also play a positive role in the recognition process.

### 2.2. Vehicle recognition and classification

Vehicle recognition and classification are essential components of FGVC field. In the context of vehicles, this entails distinguishing between closely related classes such as different car models, brands, and types, where subtle visual differences in features become crucial for accurate classification. Currently, research on vehicle recognition and classification primarily centers around three main approaches: pattern recognition based on matching method, pattern recognition based on machine learning and pattern recognition based on deep learning.

The first approach involves the identification of vehicles through license plates and vehicle tag detection using a matching method. While the license plate number and label characteristics can directly identify the vehicle’s brand and model (Psyllos et al., 2010; Huang et al., 2015), this method has a limitation: it does not encompass all the fine-grained features associated with the vehicle brand and model. Apart from the license plates and labels, vehicle lights and other textural information also bear the characteristics of the vehicle model. Relying solely on license plates and tags is insufficient. Additionally, the license plates of trucks are prone to being contaminated by dirt and dust, which leads to reduced visibility and clarity. In such scenarios, this method becomes ineffective.

The second approach involves using machine learning to classify vehicle brands and models. The traditional machine learning method comprises two steps: feature extraction and classifier classification. Fraz et al. proposed a method for recognizing vehicle brands and models based on a SIFT feature dictionary (Fraz et al., 2014). In this method, SIFT features of pictures from the training set’s vehicles were treated as “words” to create a dictionary of vehicle brands and models. However, this method necessitates extensive computation and takes a considerable amount of time to identify each image, making it unsuitable for real-time vehicle brand and model classification in practical scenarios. Abdul et al. proposed a method employing a cascade classifier (Siddiqui et al., 2016). Initially, representative features were extracted from the samples instead of using all features. Subsequently, a cascade-based SVM classifier was employed, resulting in significant improvements in real-time recognition. Biglari et al. (2019) introduced an algorithm based on the histogram of gradient directions feature and cascade classifier. Multiple vehicle brand models were trained first, followed by classification using a cascade SVM classifier, achieving an impressive classification accuracy of up to 96.78%. However, this method still requires hardware acceleration for real-time classification.

The third approach involves vehicle pattern recognition based on deep learning. Yang et al. proposed a method for recognizing vehicle brands and models based on the joint attributes of vehicles (Yang et al., 2015). This method extracts vehicle features from multiple perspectives and angles, fuses the extracted features, and performs recognition. While this method is well-suited for recognizing vehicle brands and models in complex scenes, its real-time performance is compromised due to the abundance of features. Huang et al. suggested randomly discarding certain layers during the training of ResNet to obtain a convolutional neural network with random depth (Huang et al., 2016), thereby addressing the issue of gradient vanishing caused by excessively deep networks. Fang et al. introduced a fine-grained method for recognizing vehicle brands and models (Fang et al., 2016), utilizing a CNN model to extract local and overall features of vehicles, and combining them for classification. Wang et al. (2020) proposed a method based on structural graph to learn discriminative representations for vehicle recognition. This approach first constructs a global structural graph from the features generated by a convolutional network. Then, it utilizes this structural graph as guidance to generate effective vehicle representations. Mo et al. (2020) analyzed the relationship between the number and distribution of vehicle axles and the weight limit of trucks. They proposed a circular detection method based on an improved Hough and clustering algorithm to identify the axles of trucks. Presently, most studies on deep learning for vehicle brand recognition rely on a single convolutional neural network model. However, for the intricate task of truck brand classification, a single model falls short in achieving satisfactory classification accuracy. Consequently, integrating multiple convolutional neural network models to develop a fusion model suitable for truck brand classification becomes a problem that requires resolution in this study.

## 3. Method

**Figure 1:**
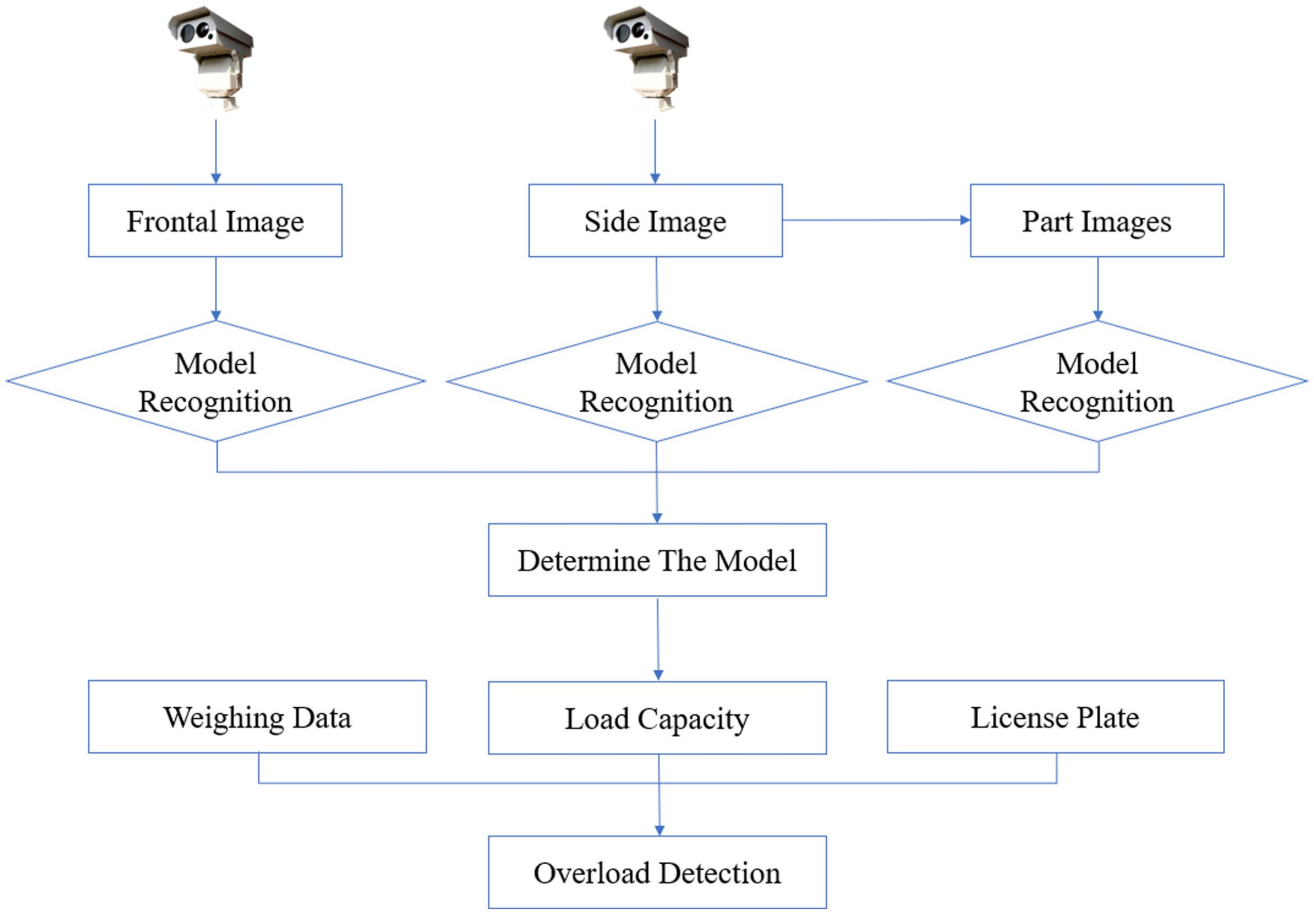
The process of the overload detection.

In typical scenarios, automatic weighing stations on highways are equipped with multiple cameras to capture frontal and side images of trucks. When utilizing these images for model recognition, the initial step involves utilizing the frontal image (front view) for identification. Analyzing the frontal image allows for the determination of the truck’s model. Additionally, the side image is utilized to enhance accuracy in identifying the frontal view. The side image provides supplementary perspectives and details, thereby improving the accuracy of frontal view recognition. Moreover, the side image enables the segmentation of the truck into multiple parts, further refining model recognition precision. Through comprehensive analysis of both frontal and side images, we can achieve more accurate truck identification and conduct additional analysis based on its body features. Knowing the truck’s model provides information regarding its rated load capacity. The weight measurement data obtained in the automatic weighing area enables straightforward determination of whether the truck is overloaded. Moreover, the inclusion of license plates information enables efficient monitoring and regulation by traffic authorities. Figure 1 illustrates the process described above.

### 3.1. The improved MMAL-Net

**Figure 2:**
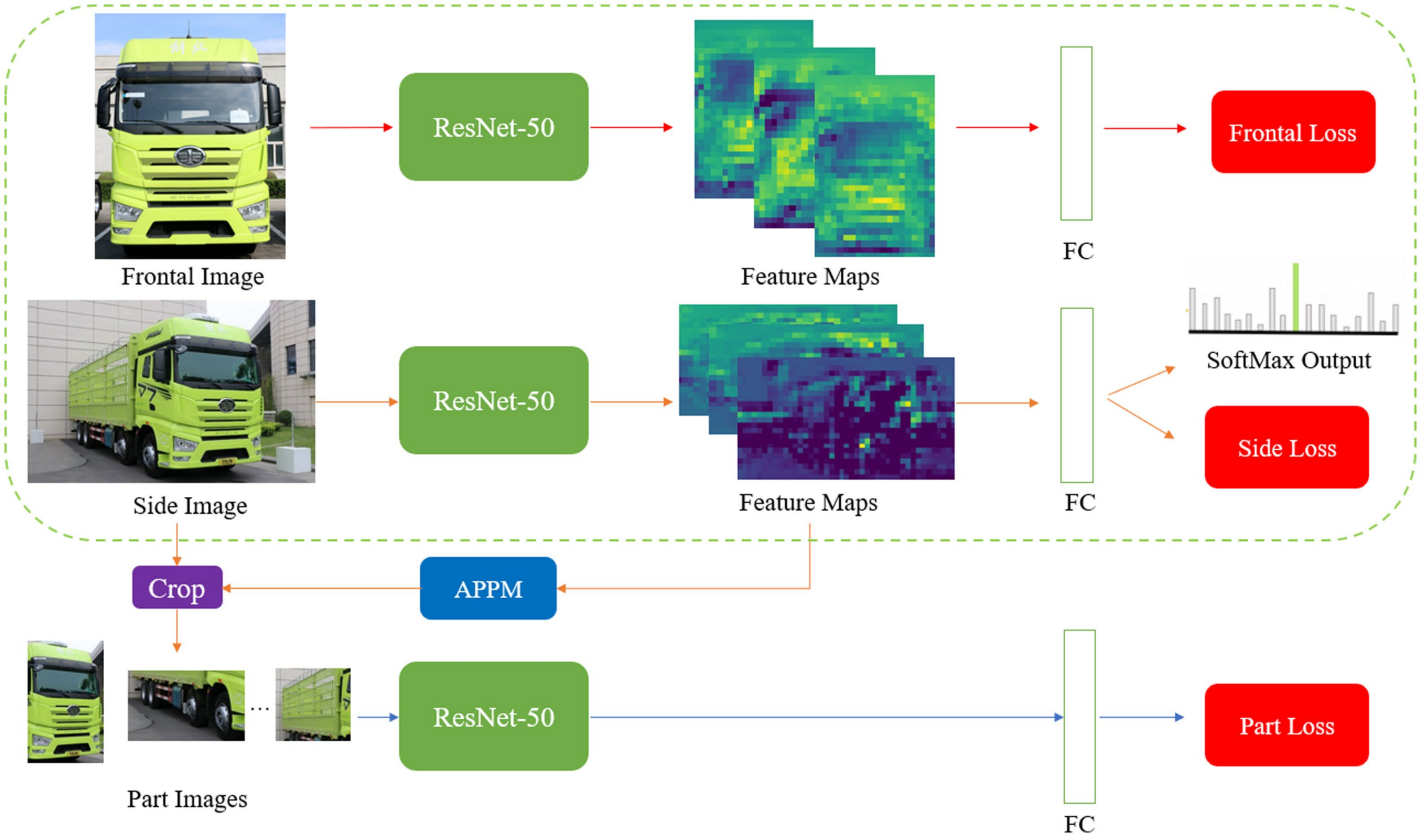
The improved MMAL-Net in the training phase.

We improved MMAL-Net (Zhang et al., 2021) and employed it for truck recognition and classification. In Figure 2, we illustrate the network architecture that was constructed during the training phase, consisting of three branches: frontal, side, and part branches. The frontal branch is responsible for recognizing and classifying frontal truck images, while the side branch receives side images and segments them into multiple parts using the Attention Part Proposal Module (APPM). The part branch, on the other hand, specializes in recognizing and classifying part images. All three branches utilize a ResNet-50 (He et al., 2016) for feature extraction and employ a Fully Connected (FC) layer for classification, employing cross-entropy loss as the classification loss function.

ResNet-50 is a CNN architecture that belongs to the ResNet family. The ResNet family of architectures was specifically developed to address the problem of vanishing gradients in deep neural networks. In ResNet-50, the numerical suffix “50” indicates that the network consists of a total of 50 layers, including convolutional layers, pooling layers, fully connected layers, and shortcut connections. The key innovation of ResNet lies in the introduction of residual or skip connections, which allow information to bypass certain layers. This enables the network to learn more effectively by facilitating the propagation of gradients during training and enabling the acquisition of deeper and more complex representations. These skip connections also mitigate the problem of degradation, wherein the accuracy of a deep network decreases as its depth increases, by facilitating the training of deeper networks. ResNet-50 has been widely utilized and has achieved significant success in various machine vision tasks, such as image classification, object detection, and image segmentation. It has proven to be a powerful architecture that has advanced the field of computer vision and deep learning.

Formulas 1, 2, and 3 represent the loss function of the three branches, respectively.


(1) $$L_{frontal}=-\log\left(P_{f}\left(c\right)\right)$$

(2) $$\begin{array}{c} L_{side}=-\log\left(P_{s}\left(c\right)\right) \end{array}$$

(3) $$\begin{array}{c} L_{part}=-\overset{N-1}{\underset{n=0}{\sum }}\log\left(P_{p\left(n\right)}\left(c\right)\right) \end{array}$$

Where c represents the ground truth label of the input image, while $P_{f}$ and $P_{s}$ denote the category probabilities obtained from the last softmax layer outputs of the frontal and side branches, respectively. $P_{p\left(n\right)}$ refers to the output of the softmax layer in the part branch that corresponds to the n th part image. N represents the total count of part images.

The total loss is defined as Formula 4:


(4) $$\begin{array}{c} L_{total}=L_{frontal}+L_{side}+L_{part} \end{array}$$

The total loss is calculated as the cumulative sum of losses from the three branches, collaborating to enhance the model’s performance during backpropagation. This enables the final converged model to generate classification predictions by considering both the global structural attributes of the object and its detailed features. During the testing phase, the part branch was excluded to minimize computational complexity, ensuring efficient prediction times for practical applications of our method.

### 3.2. APPM

**Figure 3:**
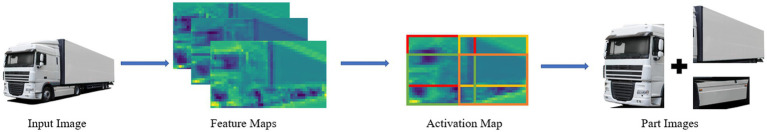
The simple pipeline of the APPM. We use red, orange, yellow and green colors to indicate the order of windows’ ${\bar{a}}_{w.}$


By analyzing the activation map A , we observed that areas with high activation values corresponded to key parts, such as the front area of the truck. To identify these informative regions, we adopted a sliding window approach inspired by object detection techniques. This approach allowed us to extract part images from windows containing relevant information. Additionally, we employed a modified version of the traditional sliding window method using a fully convolutional network, similar to the approach used in Overfeat (Sermanet et al., 2013). This method involved obtaining feature maps for different windows from the output feature map of the previous network branch. Subsequently, we aggregated the activation maps $A_{w}$ of each window along the channel dimension and computed their mean activation value ${\bar{a}}_{w}$ , as described in Formula 5. Here, $H_{w}$ and $W_{w}$ denote the height and width of a window’s feature map, respectively. We then ranked the windows based on their ${\bar{a}}_{w}$ values, with higher values indicating more informative regions, as illustrated in Figure 3.

(5) $$\begin{array}{c} {\bar{a}}_{w}=\frac{\sum_{x=0}^{W_{w}-1}\sum_{y=0}^{H_{w}-1}A_{w}\left(\mathrm{x,y}\right)}{H_{w}\times W_{w}} \end{array}$$

**Figure 4:**
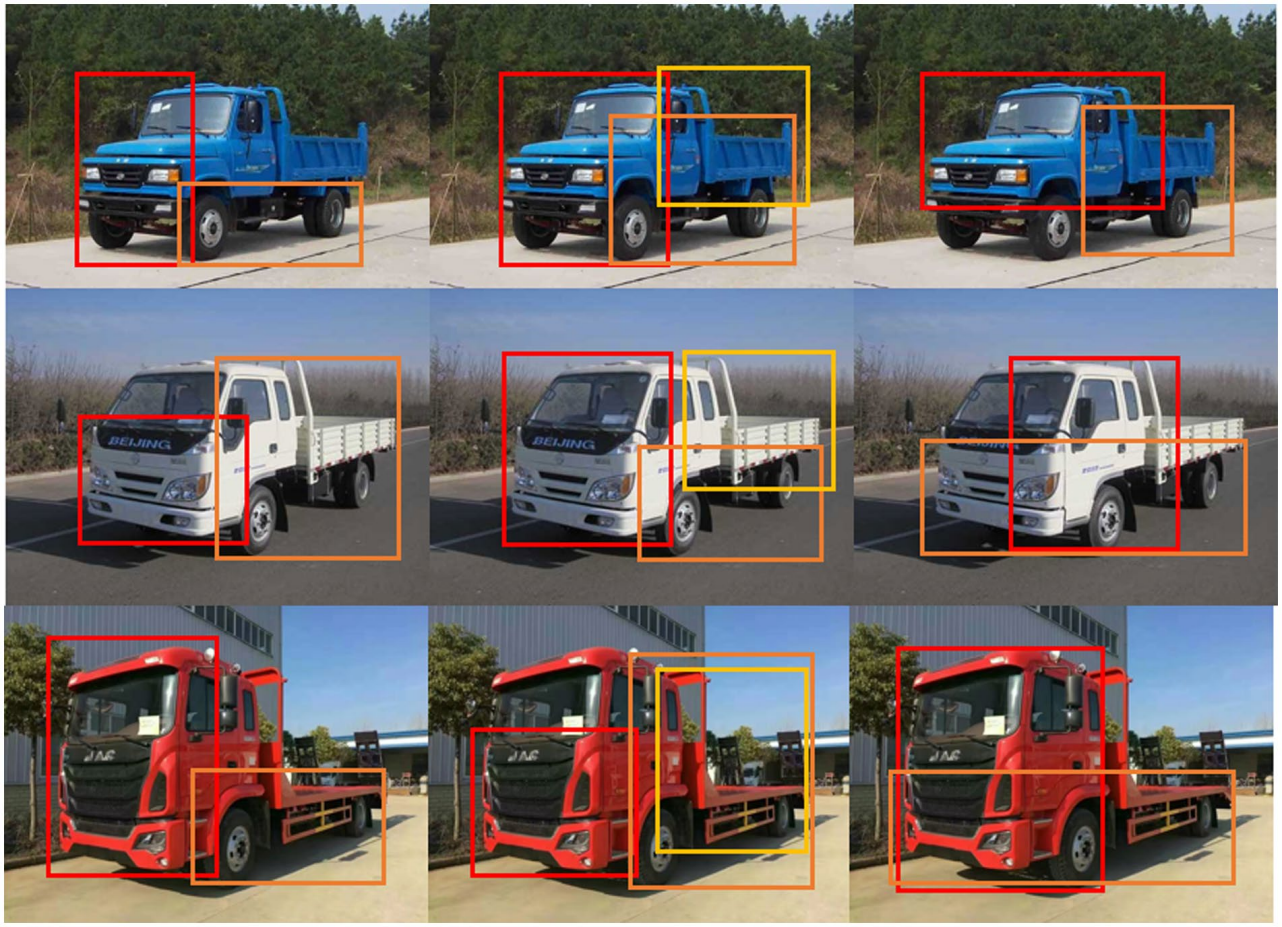
Visualization of part regions.

However, we cannot directly select the initial windows because they are often adjacent to the windows with the highest average activation values ${\bar{a}}_{w}$ and contain nearly identical parts. Nonetheless, our objective is to choose a diverse range of parts. To minimize redundancy in the regions, we employ Non-Maximum Suppression (NMS) to select a fixed number of windows as part images at different scales. The visualization of the module’s output in Figure 4 demonstrates that the proposed method effectively identifies distinct part regions with varying levels of importance. We utilize red, orange, yellow, and green rectangles to highlight the regions proposed by APPM that have the highest average activation values at various scales, with the red rectangle indicating the highest value. Figure 4 illustrates that the proposed approach captures detailed information and exhibits a more logical ordering at the same scale, thus significantly enhancing the model’s robustness to scale variations. Notably, the head region stands out as the most discriminative region for truck recognition.

## 4. Results and discussion

To validate the advantages of the enhanced MMAL-Net, we conducted an evaluation of our method on the well-established and competitive benchmark dataset, Stanford Cars (Krause et al., 2013).

In our experiments, we adopted a consistent preprocessing approach. Initially, we resized the images to dimensions of 512 × 512, serving as inputs for both the frontal and side branches. Additionally, all part images were uniformly resized to 256 × 256 for the part branch. To ensure efficient initialization, we pre-trained ResNet-50 on the widely used ImageNet dataset, allowing us to effectively obtain the network’s initial weights. Throughout both the training and testing phases, we exclusively relied on image-level labels, refraining from employing any additional annotations. Our optimization process involved utilizing SGD with specific hyperparameters: a momentum value of 0.9 and a weight decay of 0.0001. To enhance training efficiency, we employed a mini-batch size of 6, utilizing a Tesla P100 GPU for computation. For fine-tuning the learning process, we set the initial learning rate to 0.001, which we later scaled down by a factor of 0.1 after 60 epochs. This step was instrumental in facilitating smoother convergence during training. We utilized PyTorch as the foundational framework.

In the experiments, we compared the proposed method to several baseline approaches and achieved competitive results, as shown in Table 1. By comparison, we can observe that our method attains the highest accuracy 95.03%.

**Table 1:** Comparison of different methods on the Stanford Cars dataset.

Methods	Backbone	Source	Accuracy (%)
RA-CNN Fu et al. (2017)	VGGNet-19	CVPR’2017	92.5
MA-CNN Zheng et al. (2017)	VGGNet-19	ICCV’2017	92.8
NTS-Net Yang et al. (2018)	ResNet-50	ECCV’2018	93.9
MAMC Sun et al. (2018)	ResNet-101	ECCV’2018	93.0
TASN Zheng et al. (2019)	ResNet-50	CVPR’2019	93.8
DCL Chen et al. (2019)	ResNet-50	CVPR’2019	94.5
API-Net Zhuang et al. (2020)	ResNet-50	AAAI’2020	94.8
DP-Net Wang et al. (2021)	ResNet-50	AAAI’2021	94.8
SAM Shu et al. (2022)	ResNet-50	ECCV’2022	94.18
MSHQP Tan et al. (2022)	ResNet-152	TOMM’2022	94.9
The Improved MMAL-Net	ResNet-50	This paper	95.03

**Figure 5:**
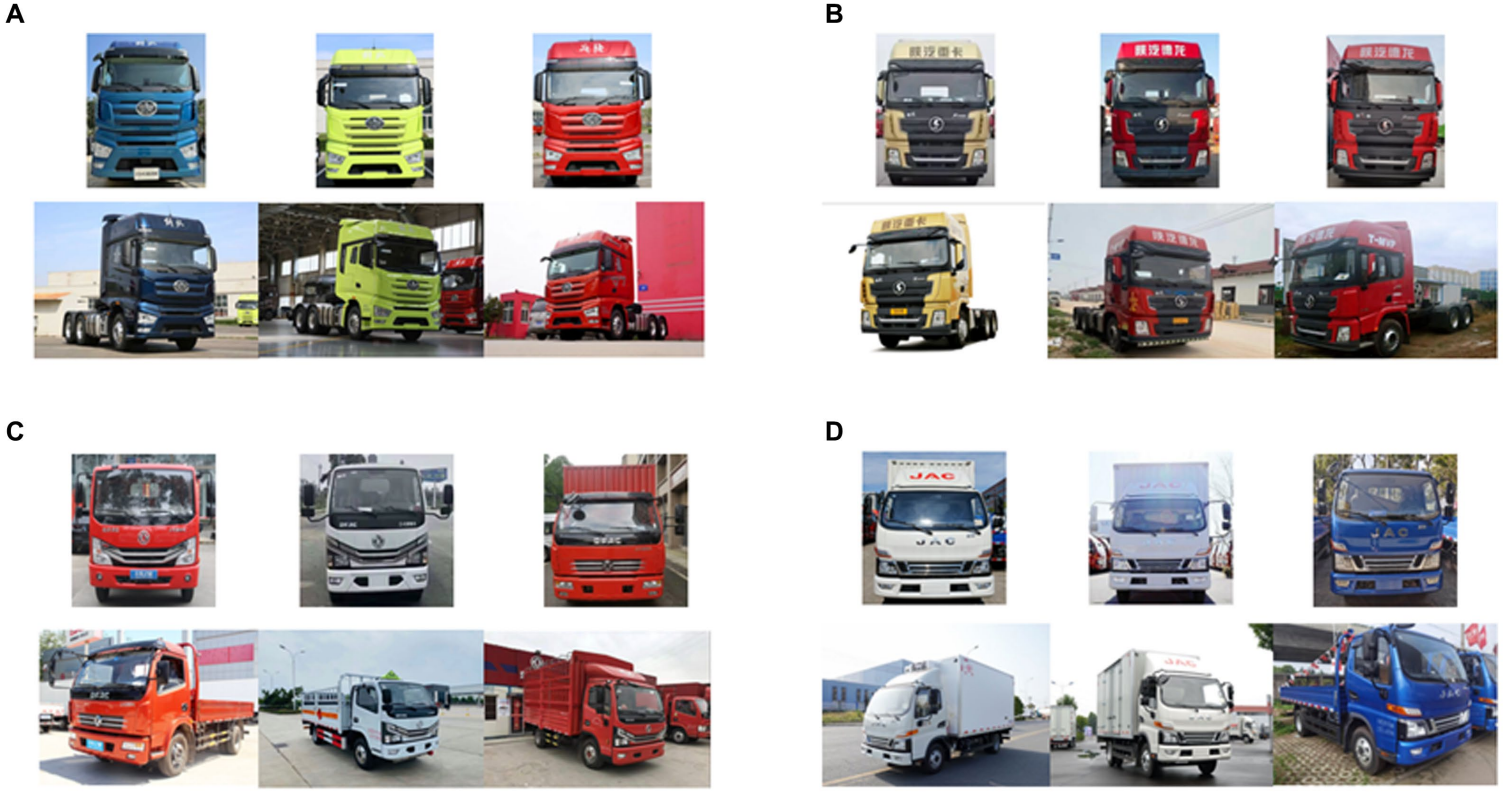
The personalized truck dataset. **(A–D)** represent FAW J7, Shaanxi Delong X3000, Dongfeng Dorica D6, and JAC Junling V6.

In practice, there is a continuous emergence of new truck models. Given their recent introduction, it becomes challenging to obtain an adequate number of instances for constructing a comprehensive dataset. Hence, we utilized a customized dataset on a smaller scale to validate the applicability and effectiveness of our method. The personalized truck dataset includes four truck models: FAW J7, Shaanxi Delong X3000, Dongfeng Dorica D6, and JAC Junling V6. FAW J7 and Shaanxi Delong X3000 are heavy-duty trucks, whereas Dongfeng Dorica D6 and JAC Junling V6 are light-duty trucks. Each truck category is composed of 50 sets of training images and 20 sets of test images and each set comprises one frontal image and one side image. An example is depicted in Figure 5.

Thereafter, the overall network structure with specific features was fine-tuned to achieve fine-grained recognition of multiple target models. Moreover, we evaluated the effect of varied training samples on the recognition performance of the intelligent identification model by testing the same dataset using different incremental levels of training data. This simulation emulated the impact of increasing the number of target truck images collected in actual scenarios on the enhancement of the recognition model’s performance.

**Figure 6:**
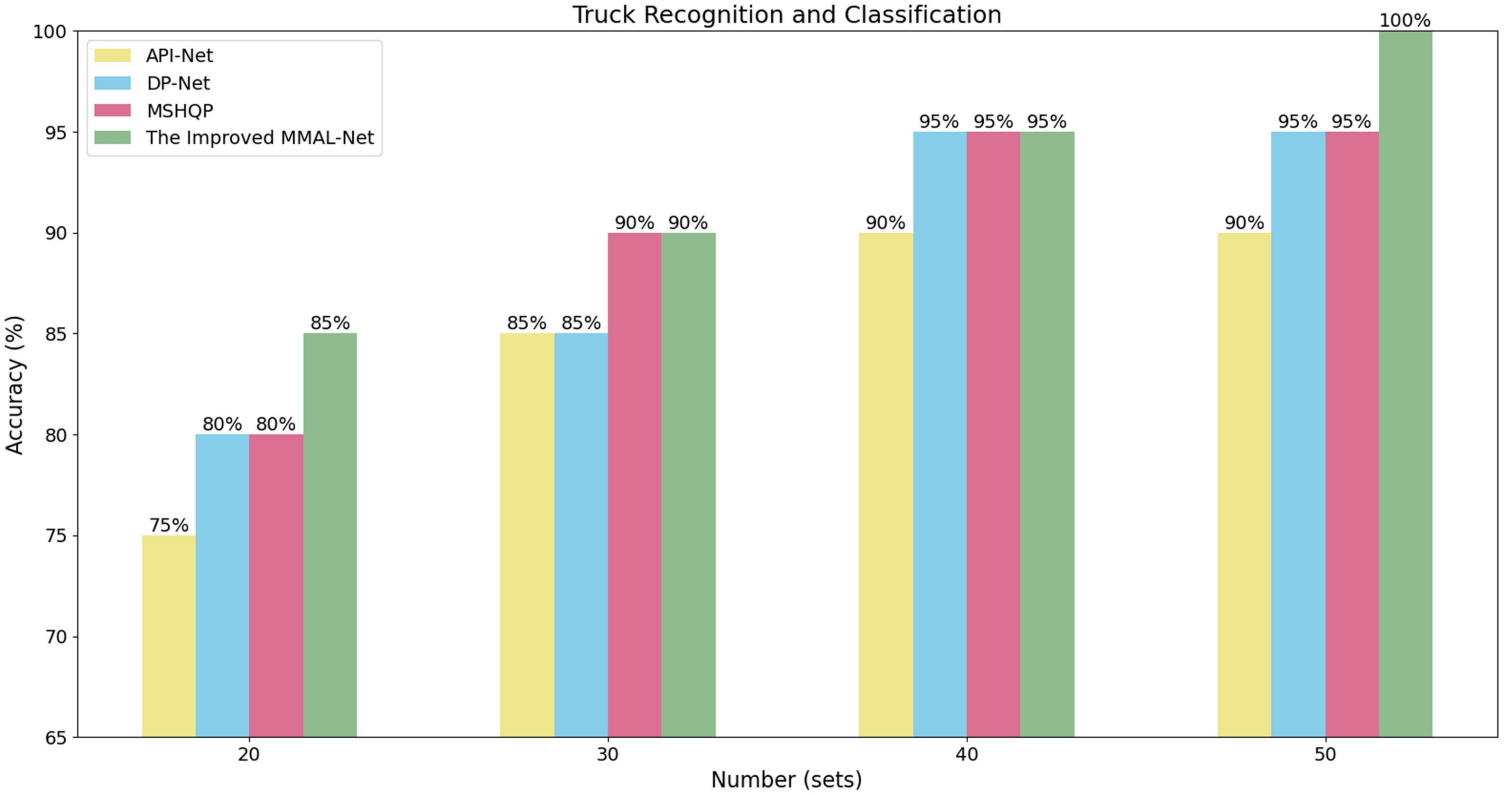
The comparison result on different number of training sets.

In our experiment, we selected sets of 20, 30, 40, and 50 images for each classifier as training datasets and used the same number of test set to compare the performance of API-Net, DP-Net, MSHQP and the improved MMAL-Net. It is worth mentioning that API-Net, DP-Net, and MSHQP were the top three performing methods in our experiments on the Stanford Cars dataset, excluding our proposed method. The results indicate that as the training data increases, the network’s ability to identify and extract features from target trucks gradually improves, suggesting that larger datasets can effectively enhance the model’s capability to extract potential features. The improved MMAL-Net exhibits comparable or superior performance to other methods across all numbers of training sets, demonstrating its superior ability to extract fine-grained features of target trucks (see Figure 6).

In our small-scale custom dataset, it is evident that the recognition accuracy reaches 85% when the training set consists of 20 sets of photos. This greatly addresses the practical issue of scarce images of a particular type of truck. The improved MMAL-Net demonstrated remarkable resilience to image quality and scene noise, as evidenced by its recognition accuracy of 100% when trained on a dataset comprising 50 sets of samples. This noteworthy achievement further supports the superior performance of the enhanced network.

**Figure 7:**
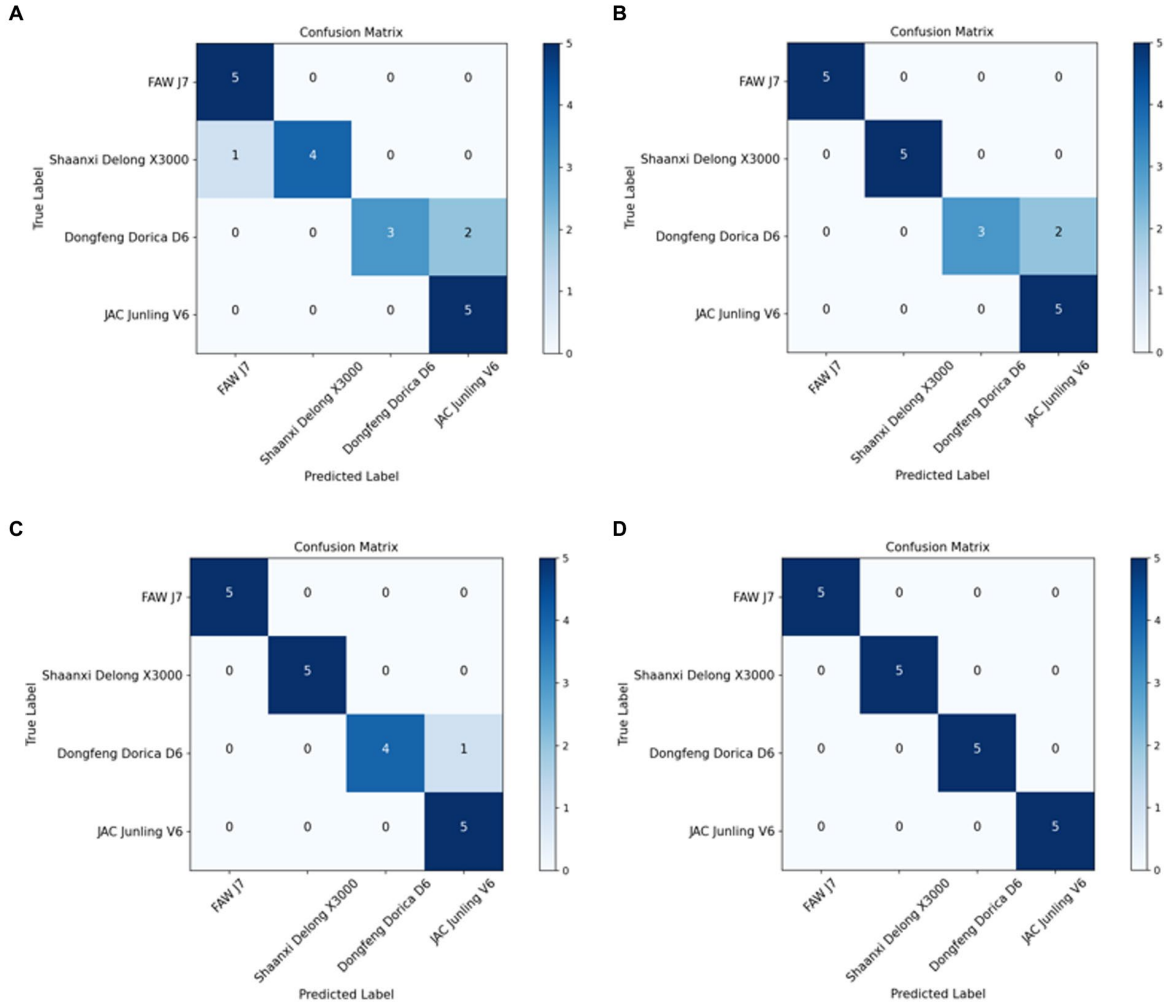
Confusion matrices of the improved MMAL-Net. **(A–D)** represent 20, 30, 40 and 50 sets of images, respectively, which are used as the training dataset.

Confusion matrices in Figure 7 illustrate the test results of the improved MMAL-Net. At a training set size of 40 sets of images, the improved MMAL-Net had a single misclassification on the test set, misclassifying a Dongfeng Dorica D6 as a JAC Junling V6. However, at a training set size of 50 sets, all classifications were accurate. The improved MMAL-Net accurately classified heavy-duty trucks, avoiding misclassification as light-duty trucks. Similarly, it correctly identified light-duty trucks without misclassification as heavy-duty trucks. This is crucial because misidentifying an overloaded light-duty truck as a heavy-duty truck can result in undetected overweight issues, thus posing safety concerns.

## 5. Conclusion

This paper introduces a method for precise identification of truck models. In our experimental evaluation, this method achieved an accuracy of 95.03% on the competitive benchmark dataset, Stanford Cars. Furthermore, it achieved an accuracy of 100% on our custom truck dataset. When integrated with weighing and license plates systems, it can be applied in highway automatic weighing stations to determine if a truck is overloaded. By providing accurate truck information, this method contributes to freight management, transportation safety, and highway planning, thereby fostering the development of the logistics industry and improving traffic safety. However, the accuracy of truck model recognition may decrease in real-world scenarios due to the reduced data quality. Consequently, future research will focus on addressing this issue, with specific emphasis on long-distance shooting conditions.

## Data availability statement

The raw data supporting the conclusions of this article will be made available by the authors, without undue reservation.

## Author contributions

JiaS contributed to conception and design of the study. JiaS and JinS organized the methodology. JiaS and ZY performed the statistical analysis. JiaS wrote the first draft of the manuscript. JiaS, ZG, and YS contributed to the visualization of the results. JiaS and LL contributed to the supervision of the manuscript. All authors contributed to the article and approved the submitted version.

## Funding

This research was supported by National Key R&D Program of China (Grant no. 2021YFB3300503).

## Conflict of interest

The authors declare that the research was conducted in the absence of any commercial or financial relationships that could be construed as a potential conflict of interest.

## Publisher’s note

All claims expressed in this article are solely those of the authors and do not necessarily represent those of their affiliated organizations, or those of the publisher, the editors and the reviewers. Any product that may be evaluated in this article, or claim that may be made by its manufacturer, is not guaranteed or endorsed by the publisher.
